# Cortical 3-hinges could serve as hubs in cortico-cortical connective network

**DOI:** 10.1007/s11682-019-00204-6

**Published:** 2020-01-16

**Authors:** Tuo Zhang, Xiao Li, Xi Jiang, Fangfei Ge, Shu Zhang, Lin Zhao, Huan Liu, Ying Huang, Xianqiao Wang, Jian Yang, Lei Guo, Xiaoping Hu, Tianming Liu

**Affiliations:** 1 School of Automation, Northwestern Polytechnical University, #127, West Youyi Road, Xi’an, 710072 Shaanxi China; 2 The Clinical Hospital of Chengdu Brain Science Institute, MOE Key Lab for Neuroinformation, School of Life Science and Technology, University of Electronic Science and Technology of China, Chengdu, China; 3 Cortical Architecture Imaging and Discovery Lab, Department of Computer Science and Bioimaging Research Center, The University of Georgia, Athens, GA USA; 4 College of Engineering, The University of Georgia, Athens, GA USA; 5 Radiology Department of the First Affiliated Hospital, Xi’an Jiaotong University, Xi’an, China; 6 The Key Laboratory of Biomedical Information Engineering, Ministry of Education, Department of Biomedical Engineering, School of Life Science and Technology, Xi’an Jiaotong University, Xi’an, China; 7 Department of Bioengineering, University of California Riverside, Riverside, CA USA

**Keywords:** Gyral hinges, Structural connectome, Functional network, Connective hub

## Abstract

Mapping the relation between cortical convolution and structural/functional brain architectures could provide deep insights into the mechanisms of brain development, evolution and diseases. In our previous studies, we found a unique gyral folding pattern, termed a 3-hinge, which was defined as the conjunction of three gyral crests. The uniqueness of the 3-hinge was evidenced by its thicker cortex and stronger fiber connections than other gyral regions. However, the role that 3-hinges play in cortico-cortical connective architecture remains unclear. To this end, we conducted MRI studies by constructing structural cortico-cortical connective networks based on a fine-granular cortical parcellation, the parcels of which were automatically labeled as 3-hinge, 2-hinge (ordinary gyrus) or sulcus. On human brains, 3-hinges possess significantly higher degrees, strengths and betweennesses than 2-hinges, suggesting that 3-hinges could serve more like hubs in the cortico-cortical connective network. This hypothesis gains supports from human functional network analyses, in which 3-hinges are involved in more global functional networks than ordinary gyri. In addition, 3-hinges could serve as ‘connector’ hubs rather than ‘provincial’ hubs and they account for a dominant proportion of nodes in the high-level ‘backbone’ of the network. These structural results are reproduced on chimpanzee and macaque brains, while the roles of 3-hinges as hubs become more pronounced in higher order primates. Our new findings could provide a new window to the relation between cortical convolution, anatomical connection and brain function.

**Electronic supplementary material:**

The online version of this article (10.1007/s11682-019-00204-6) contains supplementary material, which is available to authorized users.

## Introduction

Cerebral cortical convolution patterns have been shown to be correlated with brain structural connective patterns and brain functions to a certain extent (Van Essen 1997; Zilles et al. 1997; Thompson et al. 2004; Fischl et al. 2007; Nordahl et al. 2007; Bullmore and Sporns 2009; Honey et al. 2010). The studies of such relationships could provide useful insights into the mechanisms of brain development, evolution and abnormality (Zilles et al., 1988; Roth and Dicke, 2005; Hilgetag and Barbas 2006; Fischl et al. 2007; Dubois et al. 2008; Giedd and Rapoport 2010; Honey et al. 2010; Holland et al. 2015). For example, it has been demonstrated that cortical convolution patterns can be used as predictors of primary and secondary Brodmann areas (BAs) (Fischl et al. 2007), the boundaries between which were determined by the considerable changes of cyto- and myelo- architectures. Indeed, cortex can be further decomposed into finer-granular basic morphological patterns, such as gyri and sulci. It was found that gyri and sulci were significantly different in their cyto-architecture (Connolly 1950; Richman et al. 1975), myelo-architecture (Rakic 1984; Van Essen 1997; Hilgetag and Barbas 2005) and even on a genetic basis (Götz and Huttner 2005; Stahl et al. 2013; Zeng et al. 2015). On this basis, a school of studies focused on the relationship between these two folding patterns and axonal wiring diagram (Van Essen 1997; Hilgetag and Barbas 2006; Xu et al. 2010; Chen et al. 2013; Budde and Annese 2013; Zhang et al. 2014). For example, some reports suggested that gyri were generated by the tension on axons which pulls the cortices closer (Van Essen 1997; Hilgetag and Barbas 2006). In other studies (Xu et al. 2010; Nie et al. 2012; Budde and Annese 2013; Zhang et al. 2014), gyri were observed to be connected by axons with greater density than sulci at different scales while being spatially further away from each other. There are other studies suggest that a superficial axonal system exist at the border of white matters and gray matters and could impede the detection of axonal connections, especially in sulcal regions (Reveley et al. 2015). In spite of the debate, the consensus is that gyro-sulcal patterns are closely related to axonal connective patterns, and their relation to the brain’s structural and functional architectures can be further inferred. For example, in the tension hypothesis (Van Essen 1997), axons pull the cortices closer to reduce the cost of information transit between the cortical pairs. In another hypothesis (Nie et al. 2012), gyri were connected by denser axons and could serve as information gathering and distributing centers (Deng et al. 2014; Jiang et al. 2015), such that the information transit cost is reduced in a global manner.

**Fig. 1:**
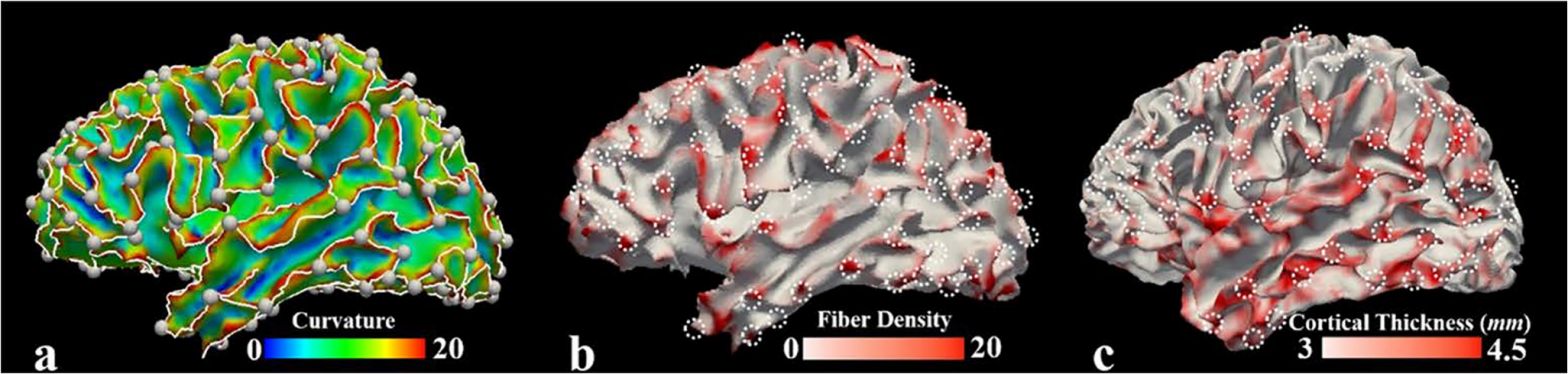
One subject is selected from the Human Connectome Project (Van Essen et al. 2013, HCP for short) dataset to show gyral hinges, fiber density and cortical thickness. **a** White bubbles indicate the locations of 3-hinges. The white curves represent gyral crest lines. The surface is color-coded with surface curvature; **b** Fiber termination density map. Deterministic streamline fibers are estimated from dMRI data. Density is defined as the numbers of fibers passing through a unit area (1*mm*^*2*^) of the surface; **c** Cortical thickness map, retrieved from the HCP dataset. The surfaces in **a** and **b** are reconstructed from the FA map of dMRI data. The one in **c** is grayordinate white matter surfaces in HCP datasets. Dashed circles in **b** and **c** highlight the locations of the same 3-hinges as those in **a**

These abovementioned cortical-area-based and gyro-sulcal-pattern-based works demonstrated that the convolution pattern of cerebral cortex is a multi-scale concept. Our recent investigations continued along this line and showed a possibility that gyral patterns can be further sub-divided. It is noted that we limited our interests in the comparison between folding patterns within gyral regions, because the contrast between different gyral folding patterns could possibly be less biased by the limitation of diffusion Magnetic Resonance Imaging (dMRI) tractography (Van Essen et al. 2014). In Li et al. 2010, a novel cortical folding pattern in gyral region was defined as a gyral hinge, which is the conjunction of gyri coming from multiple directions (white bubbles in Fig. 1a show the locations of gyral hinges). As gyral hinges with more than 4 spokes are rarely seen (Li et al. 2010), our previous and current studies focused on the ones with 3 spokes, termed 3-hinges. The uniqueness of these 3-hinges were progressively uncovered by a series of our recent works (Li et al. 2010; Yu et al. 2013; Chen et al. 2014; Jiang et al. 2015; 2018; Li et al. 2017; Ge et al. 2017): 3-hinges have thicker cortices (Fig. 1c, Li et al. 2010), stronger structural connections by means of dMRI streamline counting (Fig. 1b, Ge et al. 2017) and more pronounced structural connective diversities (Li et al. 2010; Yu et al. 2013; Chen et al. 2014; Li et al. 2017). These observations significantly contrast those on ordinary gyri, termed 2-hinges.

Although these studies have shown the possibility of gyral sub-division, the role that 3-hinges play in brain connective architecture still remains elusive. For example, 3-hinges tend to have stronger connections than other gyral regions (Ge et al. 2017), but it is unknown whether these connections are restricted to one cortical area to simply enhance the information transit efficiency or dispersed to multiple regions to make 3-hinges information segregators. To answer these questions, we reconstructed a cortico-cortical connective network to investigated the graphic metrics of 3-hinges in this work, and studied how 3-hinges contrast with their 2-hinge counterparts.

Specifically, we adopted a home-made toolkit (Chen et al. 2017) to automatically extract gyral crest lines and detect all 3-hinges locations on the entire cortical surface of an individual brain. Then, the cortical surface was parcellated to dense patches of equal area, and were used as nodes to estimate structural cortico-cortical connections and networks. These patches were labeled as 3-hinge ones, 2-hinge ones or sulcal ones, according to their locations. We only focused on the comparison between 3-hinges and 2-hinges in this work. Graphic metrics were computed to investigate the difference between the two folding patterns. All results based on structural network analyses lead to a novel hypothesis that 3-hinges could be more like hubs than 2-hinges in cortico-cortical connective networks, because of their significantly larger nodal degrees, strength and betweenness. This hypothesis gains supports from human functional analyses (Jiang et al. 2015), in which 3-hinges were involved in more global functional networks than 2-hinges. Moreover, 3-hinges tend to serve as ‘connector’ hubs between cortical communities rather than ‘provincial’ hubs within the communities, and they account for a dominant proportion of nodes in the ‘backbone’ of the cortico-cortical network. These results based on structural networks are reproduced on macaque and chimpanzee brains, while the roles of 3-hinges as hubs become more pronounced from macaque brains to human brains.

In general, these hypotheses provide a new insight into the relation between cortical convolutions, anatomical connections and brain functions, and could provide new clues to future studies of the brain development, evolution and diseases.

## Materials and methods

### Dataset description

#### Structural imaging data of human brains

In total, 64 human brains from the Q1 release of WU-Minn Human Connectome Project (HCP) consortium (Van Essen et al. 2013) were used. For T1-weighted structural MRI, the imaging parameters are: TR = 2400 *ms*, TE = 2.14 *ms*, flip angle = 8 *deg*, image matrix = 260 × 311 × 260 and resolution = 0.7 × 0.7 × 0.7 *mm*^3^.

Diffusion-weighted MRI (dMRI) data was collected with spin-echo EPI sequence. The imaging parameters are: TR = 5520 *ms*, TE = 89.5 *ms*, flip angle = 78 *deg*, FOV = 210 × 180 *mm*^*2*^, matrix = 168 × 144, resolution = 1.25 × 1.25 × 1.25 *mm*^3^, echo spacing = 0.78 *ms*. Particularly, a full dMRI session includes 6 runs, representing 3 different gradient tables, with each table acquired once with right-to-left and left-to-right phase encoding polarities, respectively. Each gradient table includes approximately 90 diffusion weighting directions plus 6 *b* = 0 *s/mm*^2^ acquisitions interspersed throughout each run. Diffusion weighted data consists of 3 shells of *b* = 1000, 2000, and 3000 *s/mm*^2^ interspersed with an approximately equal number of acquisitions on each shell within each run.

#### Functional imaging data of human brains

Task fMRI data from Q1 release of WU-Minn Human Connectome Project (HCP) consortium were used (Van Essen et al. 2013). The acquisition parameters of task fMRI data are as follows: 90 × 104 matrix, 72 slices, in-plane FOV = 208 × 180 *mm*^*2*^, 2.0 *mm* isotropic resolution and 1200 time points, TR = 0.72 *s*, TE = 33.1 *ms*, flip angle = 52 *deg*, BW =2290 *Hz/Px*. Dataset descriptions can be found in Glasser et al. 2013. Task designs can be found in Barch et al. 2013.

#### Structural imaging data of chimpanzee brains

In this study, MRI data from 16 chimpanzees are used. All the chimpanzee subjects were members of a colony in the Yerkes National Primate Research Center (YNPRC) at Emory University in Atlanta, Georgia. All imaging studies were approved by the institutional animal care and use committee (IACUC) of Emory University. The anatomical MRI scans were performed on a Siemens 3 T Trio scanner with a standard birdcage coil. Foam cushions and elastic straps were used to minimize head motion.

T1-weighted MRI data were acquired with a 3D magnetization-prepared rapid gradient echo (MPRAGE) sequence for all participants. Subjects scanned used the SS-EPI (single-shot double spin-echo echo planar imaging) sequence. The scan protocol is as follows: TR = 2400 *ms*, TE = 4.13 *ms*, flip angle = 8 *deg*, image matrix = 256 × 256 × 192 and resolution = 0.8 × 0.8 × 0.8 *mm*^3^, with 2 averages.

The parameters used for dMRI data acquisition are as follows: diffusion-weighting gradients applied in 60 directions with a *b*-value of 1000 *s/mm*^2^, TR/TE of 5900/84 *ms*, FOV of 129 × 230 *mm*^2^, matrix size of 72 × 128, resolution of 1.8 × 1.8 × 1.8 *mm*^3^, 41 slices with no gaps, covering the whole brain. Six *b* = 0 *s/mm*^2^ acquisitions were also acquired with matching imaging parameters.

#### Structural imaging data of macaque brains

MRI scans from 20 macaques were used. All the macaque subjects were members of a colony at YNPRC. All MRI scans were approved by IACUC of Emory University. The anatomical MRI scans were performed on a Siemens 3 T Trio scanner with a standard knee coil. To minimize head motion, foam cushions and elastic straps were used during the scan. Particularly, a specially designed holding device was used to stabilize macaque’s head during scanning with 2 plastic screws anchoring in the macaque’s ear canals tightly.

The T1-weighted MRI data was acquired with a 3D MPRAGE sequence. The scan protocol is as follows: TR = 2500 *ms*, TE = 3.49 *ms*, flip angle = 8 *deg*, image matrix = 256 × 256 × 192 and resolution = 0.5 × 0.5 × 0.5 *mm*^3^, with 3 averages.

The parameters used for dMRI acquisition are as follows: diffusion-weighting gradients applied in 60 directions with a *b*-value of 1000 *s/mm*^2^, TR/TE of 6970/104 *ms*, FOV of 141 × 141 *mm*^2^, matrix size of 128 × 128, resolution of 1.1 × 1.1 × 1.1 *mm*^3^, 41 slices with no gaps, covering the whole brain. Five *b* = 0 *s/mm*^2^ acquisitions were acquired with matching imaging parameters.

### Data Preprocessing

Skull removal, motion correction and eddy current correction in FSL (Andersson and Sotiropoulos 2016; Jenkinson et al. 2012) were performed on dMRI data. Next, the model-free generalized Q-sampling imaging (GQI) method (Yeh et al. 2010) in DSI Studio (Yeh et al. 2013) was adopted to calculate spin distribution function (SDF), an orientation distribution function of diffusing spins. The deterministic streamline tracking algorithm (Yeh et al. 2013) in DSI Studio was used to reconstruct 4 × 10^4^ fiber tracts for each subject using the default fiber tracking parameters (max turning angle: 60^○^, streamline length: 30 *mm*-300 *mm*, step length: 1 *mm*, quantitative anisotropy threshold: 0.2). A white matter cortical surface was reconstructed based on the tissue-segmentation result from T1-weighted MRI data via Freesurfer (Dale et al. 1999; Fischl et al. 1999a, 2002). To transpose the surface from T1-weighted MRI space to dMRI space, we firstly used a cascade of the linear registration method, FLIRT (Jenkinson et al. 2002), and the nonlinear registration method, FNIRT (Andersson et al. 2010; Jenkinson et al. 2012), to register T1-weighted MRI to the dMRI FA map. Then, the linear transformation and the nonlinear warp field were applied to the surfaces via Connectome Workbench.^1^

Human task fMRI data in the HCP dataset have been preprocessed by the minimal preprocessing pipelines (Glasser et al. 2013) upon their release. The preprocessed fMRI signals have been mapped to the standard grayordinate surface space for each subject. The grayordinate surface was registered to the warped T1-weighted white matter surface (which has been warped to dMRI space in the previous step) via surface registration in Freesurfer (Fischl et al. 1999b), such that all data modalities for each human individual were in the same space. More details about the fMRI signal preprocessing are referred to (Glasser et al. 2013; Jiang et al. 2015).

### Identification of 3-hinges

**Fig. 2:**
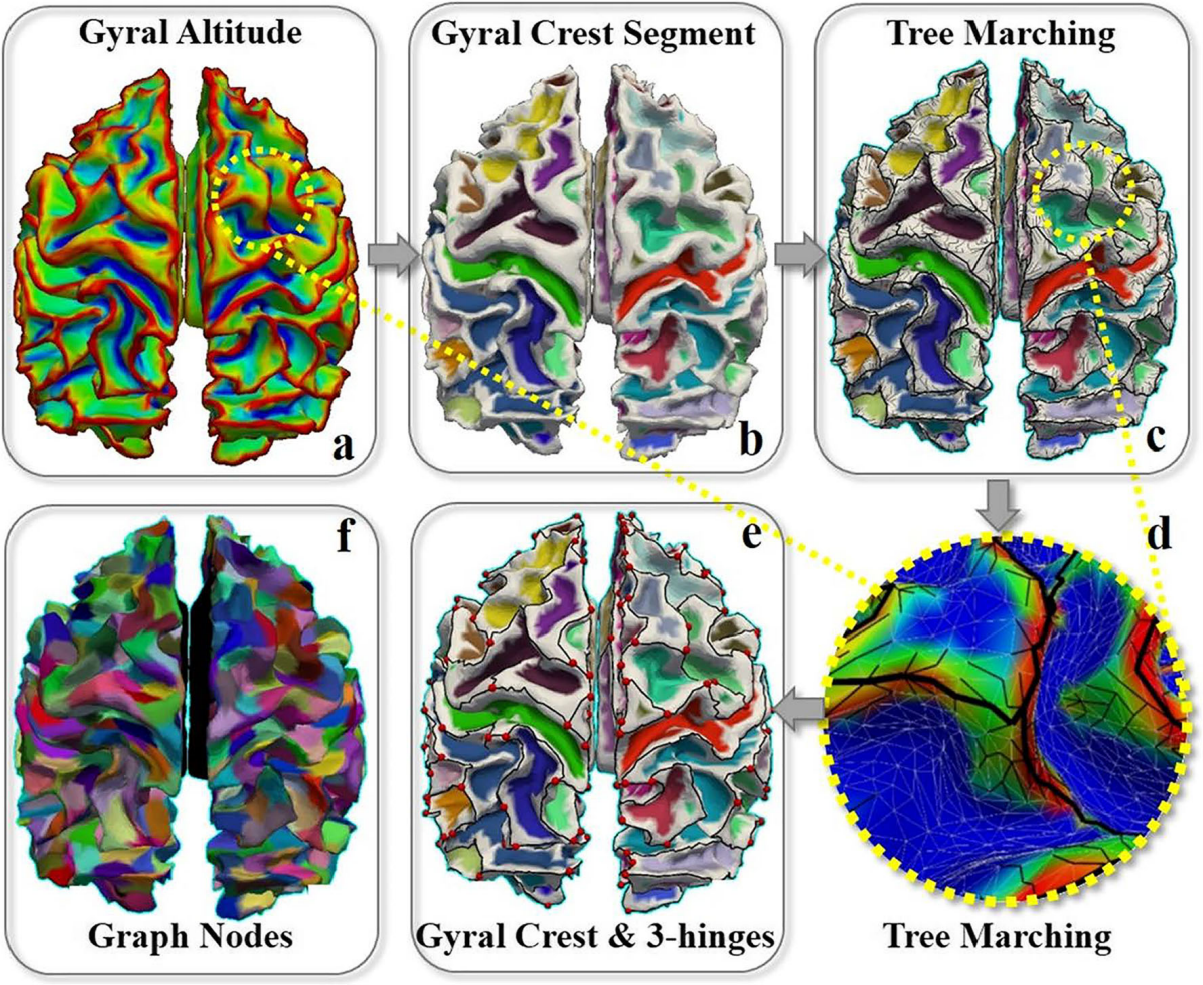
**a** White matter cortical surface mapped by gyral altitude map. Red regions have high altitude while blue regions have low altitude; **b** segmentation of gyral crests (white) from sulcal basins (labeled by different colors); **c** construction of tree structures on gyral crests (black curves); **d** an magnification view of the tree structures on gyral crests. Sulcal basins are color-coded in blue. Gyral crests are mapped with gyral altitudes. Thick black curves are the main trunks while the thin ones are branches; **e** gyral crest lines, the main trunks (black curves), and gyral hinges (red dots) on gyral crests; **f** the entire cortices are parcellated to 1000 patches of equal area (500 for each hemisphere), which are used as graph nodes for structural connective matrix

To automatically identify the locations of 3-hinges, we adopted our recently developed pipeline (Chen et al. 2017). To be self-contained, we present a summary of this pipeline, which includes the following four key steps (Fig. 2):

1. ***Gyral altitude mapping:*** The gyral altitude was defined as the movement of vertices from their original locations to the “mid-surface”, which is a mid-line that separates gyri from sulci (Fischl et al. 1999a). We mapped gyral altitudes to surface vertices. The vertices that are above the “mid-surface” have positive altitudes, and have negative values, otherwise (Fig. 2a).
2. ***Gyral crest segmentation:*** The watershed algorithm (Bertrand 2005) was applied to the gyral altitude map to progressively segment the gyral crests (regions above a predefined altitude level) from sulcal basins (regions below the level). In brief, the watershed algorithm ensures the successful detection of gyral crest that separates shallow water sources (sulcal basins) but is below the given altitude level (Fig. 2b). More details of watershed algorithm are referred to (Chen et al. 2017).
3. ***Construction of tree graphs:*** Firstly, we performed distance transform to the gyral crest regions. Each vertex was assigned a distance value, which was defined as the movement from the vertex of interest to the borders between gyral crest regions and sulcal basins. The vertex that is further from the border has a larger distance value. Next, we used a tree marching algorithm on this distance map to construct a tree structure (Fig. 2c and d). The root of the tree was located at the gyral crest centers that have the maximum distance values, and the branches and leaves were gradually connected to other gyral crest vertices following the descending gradients of the distance map till the crest borders were reached.
4. ***Extraction of gyral crest lines and 3-hinges:*** We pruned the redundant branches and preserved those located in the crest centers. To this end, we found the path between a leaf vertex and its nearest bifurcation vertex and deleted all vertices on this path when the path length was shorter than the predefined length threshold. The remaining main trunks were the gyral crest lines (the thicken tubes in Fig. 2d). By taking these crest lines as a gyral network, we defined vertices as gyral hinges when their degrees are more than 3. Three-hinge gyri, which have three arms, are of major interest in this work (red dots in Fig. 2e highlight their locations), because four-hinges are rarely seen.

### Graphic metrics on structural connective networks

Due to the inter-individual variations in terms of either structural connective patterns or cortical convolution patterns, we applied the following graphic analyses on each subject separately rather than on a group-mean network. To construct a structural connective network, nodes were defined first. Currently, we focused on cortico-cortical connectivities, and thus parcellated the cortical surface to 1000 patches, with 500 on each hemisphere (Fig. 2f). These patches are of grossly equal area and were used as the nodes for the network. The connective strength between two given patches are defined as the number of the deterministic streamline fibers that pass both of them (Van Den Heuvel and Sporns 2011). The weights do not need cross-subject normalization because the total fiber number is the same (4 × 10^4^) for all subjects.

It is noted that these cortical patches, or nodes, were labeled by the cortical convolution patterns. A patch, more than 50% of which is covered by sulcal basins (color regions in Fig. 2e), is defined as a sulcal patch. The remaining patches were defined as gyral patches. Among these gyral patches, 3-hinge patches were defined as those touched by 3-hinges (red dots in Fig. 2e), leaving the rest ones as 2-hinge patches.

On this connective network, we computed a variety of nodal metrics, including degree, strength, betweenness, efficiency, clustering coefficient and participation coefficient, as well as global metrics, such as *s*-core. All metrics were computed via Brain Connectome Toolkit (https://sites.google.com/site/bctnet/). Let *G* = (*V*, *E*) denote the structural network, where *V* is the set of *N* cortical patches/nodes and *E* is the set of weighted edges. Its adjacency matrix is denoted by *A*, where the element *a*_*ij*_ is 1 when there is a connection between nodes *i* and *j*. Let *W* denote the weight matrix where *w*_*ij*_ is the connective strength between nodes *i* and *j*.

The degree of node *i* is defined as the numbers of nodes connected to it. It is given by the following equation:

1 $$ {d}_i={\sum}_{j=1}^N{a}_{ij} $$

Strength is defined as:

2 $$ {s}_i={\sum}_{j=1}^N{a}_{ij}{w}_{ij} $$

Betweenness is defined as:

3 $$ {b}_i=\sum \limits_{s\ne i\ne t}\frac{\sigma_{st}^i}{\sigma_{st}} $$

where *σ*_*st*_ is the total number of the shortest paths from node *s* to node *t*. ${\sigma}_{st}^i$ is the number of these paths that travel through node *i*.

Efficiency is given by the following equation:

4 $$ {e}_i=\frac{\sum_{j,h\in N,j\ne i}{\left({w}_{ij}{w}_{ih}{\left[{p}_{jh}\left({N}_i\right)\right]}^{-1}\right)}^{1/3}}{d_i\left({d}_i-1\right)} $$

where *p*_*jh*_(*N*_*i*_) is the length of the shortest path between *j* and *h* that contains only neighbors of *i*.

Clustering coefficient is defined as (Onnela et al. 2005):

5 $$ {c}_i=\frac{2}{d_i\left({d}_i-1\right)}{\sum}_{j,k}{\left({\tilde{w}}_{ij}{\tilde{w}}_{jk}{\tilde{w}}_{ki}\right)}^{1/3} $$

where the ${\tilde{w}}_{ij}$ was scaled by the largest weight in the network, ${\tilde{w}}_{ij}={w}_{ij}/\mathit{\max}\left({w}_{ij}\right)$, and ${\tilde{w}}_{ij}{\tilde{w}}_{jk}{\tilde{w}}_{ki}$ is the product of the scaled weights on the edges of triangles attached to the *i*^*th*^ node.

Participation coefficient is defined based on a given partition of a network into modules. An optimal partition structure is a network subdivision that maximizes the number of within-module edges and minimizes the number of between-module edges. The network partition method used in this work is referred to (Newman 2006; Reichardt and Bornholdt, 2006). It is noted that the partition resolution is regulated by a parameter *γ*. It was set as the default value (*γ* = 1) such that a moderate partition resolution and the number of modules were automatically chosen. We used *m* to denote a module and *M* to denote the set of modules. Participation coefficient of node *i* is given by:

6 $$ {p}_i=1-{\sum}_{m\in M}{\left({d}_i(m)/{d}_i\right)}^2 $$

where *d*_*i*_(*m*) is the number of connections between node *i* and those in module *m*. Participation coefficient is usually used as a metric to measure if a node is densely connected within a module (provincial hub) or between modules (connector hub). Because there is not a universal threshold to differ connector hubs from provincial ones (it was suggested to be 0.5 in Van Den Heuvel and Sporns 2011, but 0.3 in Sporns et al. 2007), we only posit that a node will be more like a connector hubs if it has a higher *p*_*i*_ value.

#### *S-*core decomposition

The *s*-core of a weighted graph is its subgraph where all its connections possess a summed weight equal to or higher than *s* (Van Den Heuvel and Sporns 2011). The subgraph was obtained by iteratively pruning the edges whose weights are lower than *s*. Using this method, core level *s* can be assigned to a node if it is preserved after the *s-*level pruning.

#### Nodal strength decomposition

This decomposition was performed by deleting the nodes whose strength are less than *s* at *s*-level. By this way, densely self-connected high-degree nodes are detected with the increasing of *s*-level.

For both decomposition methods, we firstly identified the sub-network at a given *s*-level by their own definition. Then, we calculated the number of the preserved nodes that belong to a given convolution type (3-hinge or 2-hinge) and computed the ratio between the number of a convolution type and the number of all preserved nodes. By this way, ratios for 3-hinges and 2-hinges were obtained for a given *s*-level, and ratio curves for 3-hinges and 2-hinges were obtained by increasing the *s*-level. The decomposition and ratio curve generation were separately performed on each subject and group-mean ratio curves (with the cross-subject standard deviations at each *s*-level) were presented.

### Task-based functional network decomposition

In general, we performed dictionary learning method and sparse representation of grayordinate-based whole brain functional signals, such that a collection of dictionary components was obtained for each subject and each task data (Lv et al. 2014, 2015; Jiang et al. 2015). Suppose a grayordinate surface has *n* vertices. All their tfMRI signals were aggregated into a signal matrix ***X*** = [***x***_*1*_,…, ***x***_*n*_] ∈ ***R***^*t* × *n*^, where *t* is the tfMRI signal time points and *n* is the number of tfMRI signals. This matrix was factorized into an over-complete dictionary matrix ***D*** = [***d***_1_,…, ***d***_*k*_] ∈ ***R***^*t* × *k*^ and a sparse coefficient weight matrix ***α*** = [***α***_1_,…, ***α***_*n*_] ∈ ***R***^*k* × *n*^ via the online dictionary learning algorithm (Mairal et al. 2010), where *k* is the dictionary component size. *k* was predefined as 400 in our work as that in our previous works (Lv et al. 2014, 2015; Jiang et al. 2015). An original tfMRI signal ***x***_*i*_ is approximated as ***x***_*i*_ = ***D*** × ***α***_*i*_. In fact, each dictionary component ***d***_*i*_ is a time series that represents the activity of the *i*^*th*^ functional network, and the corresponding *i*^*th*^ row of ***α*** can be mapped to the grayordinate surface and represents the spatial pattern of this functional work. It is noted that this method was executed on the whole-brain fMRI signals, such that the obtained dictionary components, or functional networks, reflect the brain activity at a global scale.

Because the *i*^*th*^ column of ***α*** corresponds to the *i*^*th*^ vertex on the cortical surface, the values in this vector represent the degrees to which the *i*^*th*^ vertex is involved in the *k* functional networks. We counted the number of non-zero elements in ***α***_*i*_ (‖***α***_*i*_‖_0_) and used it as the number of functional networks that the *i*^*th*^ vertex was involved in. A larger number of non-zero elements for vertex *i* indicates the vertex is engaged with more global functional networks.

### Statistics

We investigated the differences between 3-hinges and 2-hinges in all structural graphic metrics and functional metrics. Because all metrics were separately computed for each subject, they were normalized within each subject via the *z*-score transformation. The values for a metric were separated to the 3-hinge group and the 2-hinge group over all subjects within a dataset. The *t*-test (two-sample) was performed to test if the two groups had equal mean values (the significance threshold of *α* = 0.05, uncorrected). To test if the difference between the two groups was produced by chance, we conducted permutation tests (Bassett et al. 2008). In a permutation test, a gyral patch was randomly assigned to either the pseudo ‘3-hinge’ group or the ‘2-hinge’ group. The sizes of the two pseudo groups were kept the same as the original ones in each subject. The between-group difference for each metric was computed on these pseudo groups. The between-group differences were defined as: 3-hinge minus 2-hinge, if 3-hinges have a greater mean than 2-hinges; 2-hinge minus 3-hinge, if 2-hinges have a greater mean than 3-hinges. This permutation test was repeated for 1000 times to sample the null distribution (the null hypothesis is that the observed h3 vs. h2 differences were determined by chance) for a metric. Finally, a *p* value was assigned to the metric by computing the proportion of the permutation tests, whose between-group-difference values were smaller than those on real brains (for scenarios where 3-hinges have a greater mean than 2-hinges), or greater than those on real brains (for scenarios where 2-hinges have a greater mean than 3-hinges). A significance threshold of *α* = 0.05 (uncorrected) was used.

To compare the metrics across species, another round of *z*-score transformation was performed across subjects within each species. The *t*-tests were conducted for 3-hinges and 2-hinges between human-chimpanzee, chimpanzee-macaque and human-macaque pairs under the null hypotheses that they have no cross-species difference in those metrics.

For the functional data of human subjects, we compared the mean numbers of functional networks that 3-hinges and 2-hinges are involved in. The two-sample *t*-test was used to investigate if the numbers are different between 3-hinges and 2-hinges (the null hypothesis is that 3-hinges and 2-hinges are involved in the same number of functional networks on average).

## Results

### Graphic metrics of 3-hinges in cortico-cortical connective networks of human brains

#### Structural cortico-cortical connective networks

**Fig. 3:**
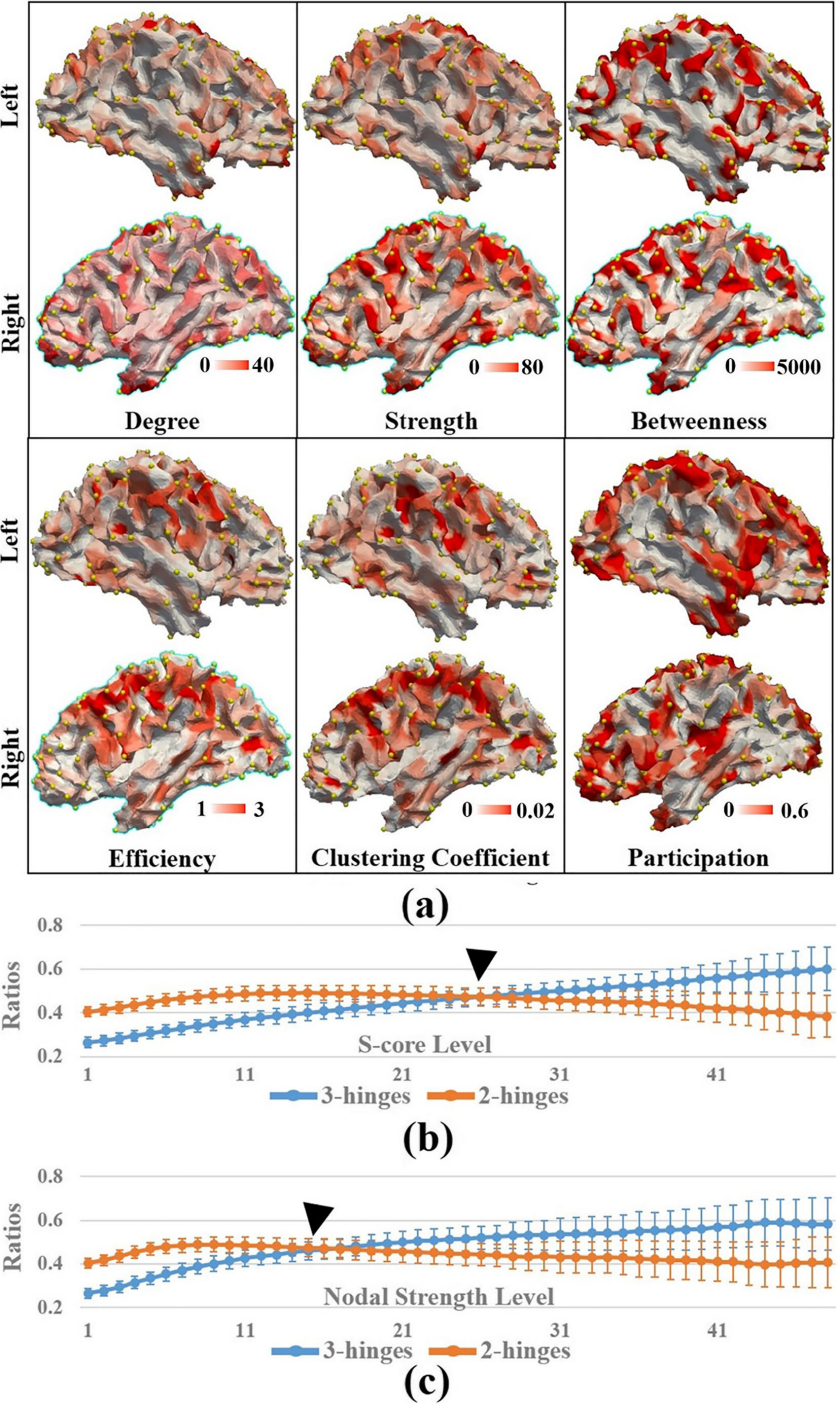
**a** Graphic metrics of structural connective network mapped to white matter cortical surface of one randomly selected human subject. For each feature, red color indicates high value and white indicates low value. The color bar for each map was individually tuned for better result visualization. Yellow bubbles highlight the locations of 3-hinges. Participation coefficients were computed based on 11 communities. **b** Ratios of numbers of preserved 3-hinges and 2-hinges at *s*-core levels, at which edges with strength less than *s* were deleted. **c** Ratios of numbers of preserved 3-hinges and 2-hinges at a nodal strength level, at which nodes with strength less than *s* were deleted. The values were averaged over subjects and the bars indicate the standard deviations. Arrow heads highlight the crossings of the two curves. Cortical parcellations at the resolution of 1000 patches were used to construct the structural connective networks

On average, 267.02 ± 28.12 3-hinges were detected on a human brain. Figure 3a gives an intuitive impression of the spatial distribution of the nodal metrics of the structural network. Locations of 3-hinge patches are highlighted by bubbles. Numeric comparisons between 3-hinges and 2-hinges are reported in Table 1. It is found that 3-hinges surpass 2-hinges in degree, strength, betweenness and participation while being surpassed by 2-hinges in efficiency and clustering coefficient. The significance of the difference between the *h3-h2* pair for each metric is demonstrated by the low *p* value via the two-sample *t*-test. It is noted that participation coefficient was computed based on a network module partition. The number of modules was automatically determined for each subject, respectively. Therefore, the module numbers are slightly different (10.42 ± 2.31) across subjects. But the major modules are consistent across subjects with regard to their spatial distribution (Fig. S1).

**Table 1:** Graphic metrics comparison among cortical folding patterns at the resolution of 1000 cortical patches on human brains

		DEG	STR	BET	EFF	CLU	PAR
Avg. ± Std.	h3	0.67 ± 1.20	0.70 ± 1.30	0.44 ± 1.51	0.09 ± 0.81	0.07 ± 0.77	0.20 ± 0.96
	h2	0.26 ± 0.93	0.20 ± 0.94	0.12 ± 0.97	0.11 ± 0.90	0.11 ± 0.89	0.12 ± 0.98
Between-Group *p*-values	h3 vs. h2	< 0.001	< 0.001	< 0.001	0.02	< 0.001	< 0.001
Permutation Tests		0.029	0.040	0.05	< 0.01	< 0.01	< 0.01

The first two rows: The mean values and standard deviations for each folding pattern. The values are averaged over subjects and cortices. h3: 3-hinge and h2: 2-hinge. The third row: *p*-values of two sample *t*-tests for the difference between folding patterns. The null hypothesis is that 3-hinges and 2-hinges have the same mean metric. The bottom row: *p*-values of permutation tests. The null hypothesis is that the observed h3 vs. h2 differences (either h3 > h2 or h3 < h2) were determined by chance. It is noted that the metrics were normalized via *z*-score transformation within each subject

*DEG* degree, *STR* strength, *BET* betweenness, *EFF* efficiency, *CLU* clustering coefficiency, *PAR* participation

We conducted the permutation tests for each metric. The *p* values were reported in the bottom row in Table 1. In general, all *p*-values are below 0.05, suggesting the graphic metric difference between 3-hinges and 2-hinges was hardly produced by chance.

In these results, 3-hinges surpass 2-hinges in degree, strength and betweenness, suggesting that 3-hinges could behave relatively more like hubs than 2-hinges. On the other hand, 3-hinges have lower efficiency and clustering coefficient. These results indicate that the neighboring nodes of 3-hinges are not heavily connected to each other, making 3-hinges possible higher-level mediators (not directly linked mediators) of network modules. This hypothesis gains further supports from the observation that 3-hinges have higher participation values, suggesting that 3-hinges could be connector hubs (inter-module) rather than provincial ones (intra-module). Fig. S2 provides a more intuitive illustration for the comparison between 3-hinges and 2-hinges in participation coefficients.

In addition, we investigated the role that 3-hinges play in a network at a global scale via the *s*-core and the nodal strength decomposition methods. Figure 3b presents the mean ratios of the 3-hinges and 2-hinges preserved at each *s*-core level, which were averaged over subjects. The initial ratios (*s*-core level = 1) are the intact ratios of 3-hinges and 2-hinges (sum of the two ratios is not equal to 1 because sulcal patches are not shown). The ratio curve of 3-hinges rises with the increase of *s*-core level. This trend is accompanied by a slow decline of the 2-hinge curve, letting 3-hinge ratio surpass it at higher levels (the black arrow head). Such a crossing is found in 3-hinge and 2-hinge ratio curves for the nodal strength decomposition as well (Fig. 3c). In a word, higher-level network cores tend to have more 3-hinges.

To test the validity of the result, we conducted a 1000-times permutation test by shuffling the node labels. In each permutation test, the ratio curves were produced based on the new node labels. We recorded the times that the 3-hinge curve surpasses the 2-hinge curve in these 1000 tests. These 1000 test results gave the null distribution, in which the chance that 3-hinge curve surpass the 2-hinge curve was far below 0.01 for both decomposition methods, demonstrating the observation in Fig. 3b and c was not produced by chance.

#### Validation from fMRI data analyses

These structure-based results in the previous section gain supports from functional analyses. For each cortical surface vertex, we have its convolution pattern label as well as the numbers of functional networks that the vertex was involved in. Therefore, we could quantitatively compare the functional network involvement between 3-hinges and 2-hinges. The mean functional network numbers (± standard deviations) for each convolution pattern averaged over cortices and subjects are shown in the left section of Table 2. It is noted that these analyses were separately performed on each task, such that the metric values are at different scales and not directly comparable across tasks. It is observed that 3-hinges are involved in more networks that 2-hinges. The significance of the difference was demonstrated by low *p*-values of *t*-tests reported in the right section of Table 2. These results suggest that 3-hinges might be more like mediators among more functional networks in contrast to 2-hinges, as is consistent with the implication from the structural results.

**Table 2:** Left: The average numbers (± standard deviations) of functional networks that a cortical folding pattern (h3: 3-hinge and h2: 2-hinge) is involved in during the task performance; Right: *P* values of two sample *t*-tests for the differences between the folding patterns in terms of the numbers of functional networks.

	Avg. ± Std.		*P*-value
	h3	h2	h3 vs. h2
EMO	83.20 ± 8.83	81.98 ± 13.71	< 0.001
LAN	142.07 ± 11.47	140.25 ± 21.47	< 0.001
MOT	129.23 ± 11.68	127.78 ± 20.07	< 0.001
SOC	126.09 ± 10.74	124.60 ± 19.32	< 0.001
WM	178.42 ± 13.09	176.15 ± 26.27	< 0.001
REL	107.99 ± 10.04	106.56 ± 17.02	< 0.001
GAM	117.11 ± 10.42	115.28 ± 18.13	< 0.001

Altogether, 400 functional networks were decomposed from all fMRI signals within each task. The null hypothesis is that 3-hinges and 2-hinges are involved in the same number of functional networks on average. The analyses were separately conducted on each task

*EMO* emotion, *LAN* language, *MOT* motor, *SOC* social, *WM* working memory, *REL* relational, *GAM* gambling

### Reproducibility on macaque and chimpanzee brains

In this section, we present the comparison between 3-hinge and 2-hinge with terms of graphic metrics on structural cortico-cortical networks. On average, 156.94 ± 29.00 and 120.65 ± 15.64 3-hinges were detected on chimpanzee and macaque brains (267.02 ± 28.12 on human brains). White matter surfaces with identified 3-hinges on the three species can be found in Fig. S3. We parcellated the cortical surface to 1000 cortical patches for the two species. The patches were labeled as either ‘3-hinge’, ‘2-hinge’ or ‘sulcus’. The numbers of the streamline fibers connecting among 3-hinges, 2-hinges and the non-cortical regions (*non* for short) are reported in Table 3. It is noted that the fiber numbers in this table were corrected by the total areas of the connected cortical patches and the total fiber number on an individual is 4 × 10^4^. Therefore, the numbers in this table are equivalent to the corrected connective strength among the three regions. It is seen that the 3-hinges have the strongest connections to non-cortical regions for all the three species. The *h3-h2* connections are the strongest for all as well. The *h3-h3*, *h3-h2* and *h3-non* connection numbers increase from macaque to human.

**Table 3:** Average numbers of streamline fibers connecting non-cortical regions (non) and the cortices of two cortical folding patterns: 3-hinges (h3) and 2-hinges (h2)

	h3	h2	non
Human			
h3	7.49 ± 0.78	11.59 ± 0.75	2.14 ± 0.43
h2	11.59 ± 0.75	7.16 ± 0.47	1.60 ± 0.34
non	2.14 ± 0.43	1.60 ± 0.34	0.87 ± 0.31
Chimpanzee			
h3	4.47 ± 1.30	8.14 ± 1.67	2.51 ± 0.80
h2	8.14 ± 1.67	5.10 ± 0.97	2.29 ± 0.55
non	2.51 ± 0.80	2.29 ± 0.55	1.12 ± 0.36
Macaque			
h3	4.85 ± 0.55	6.75 ± 0.86	4.01 ± 1.12
h2	6.75 ± 0.86	3.28 ± 0.95	3.28 ± 0.93
non	4.01 ± 1.12	3.28 ± 0.83	2.91 ± 1.22

The numbers were corrected by connected cortical areas and were averaged over subjects within each species. The total number of streamline fibers for each subject is 4 × 10^4^

**Fig. 4:**
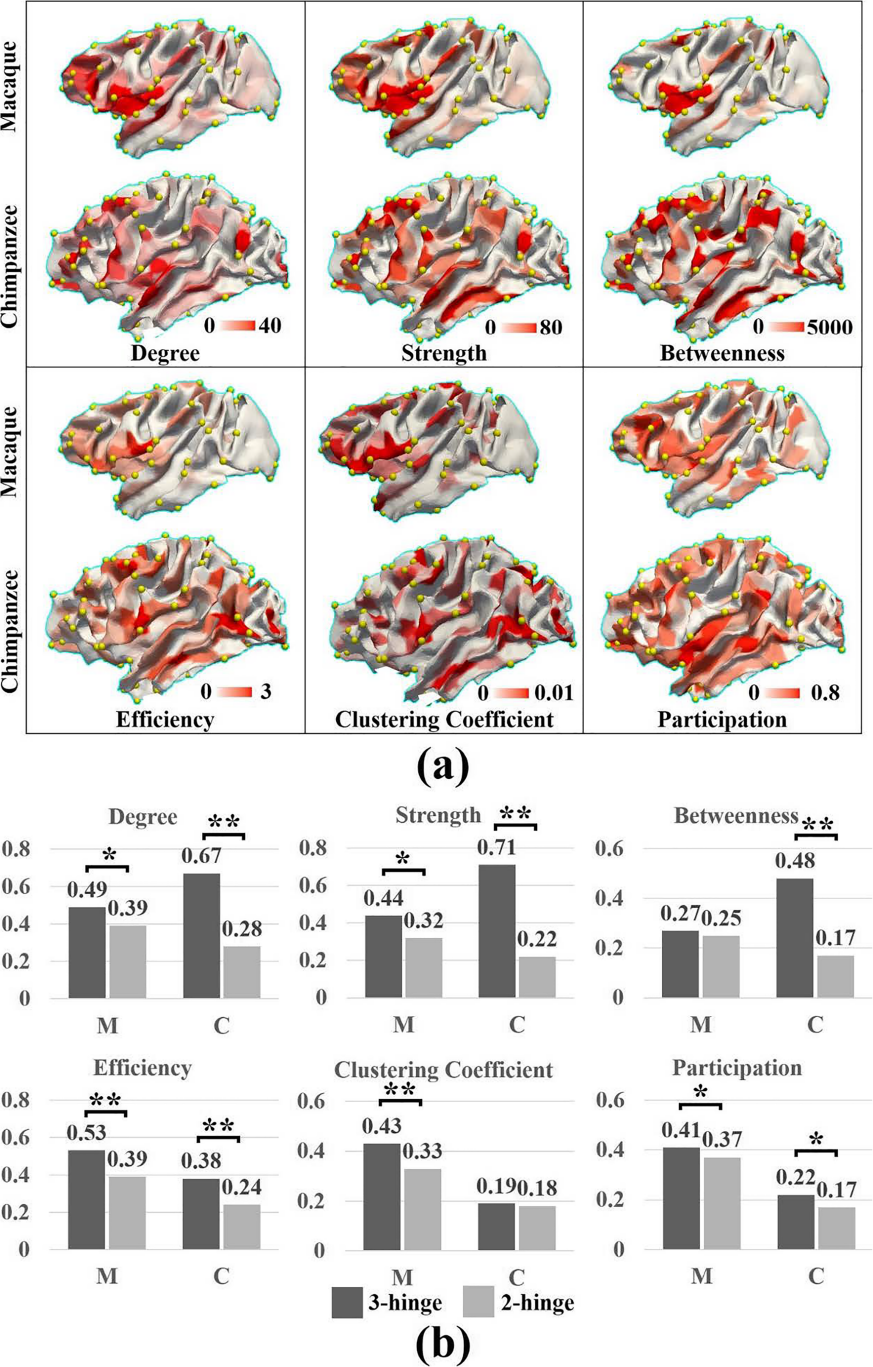
**a** Graphic metrics of structural connective networks mapped to white matter cortical surfaces of the two primates (macaque and chimpanzee). Left hemispheres were used in this illustration, and right hemispheres were found in Fig. S4. For each metric, red color indicates high value and white indicates low value. The color bar for each map was individually tuned for better result visualization. Thus, the color maps in this figure are not comparable across species. For each species, one subject is randomly selected as the illustrative example. Yellow bubbles highlight the locations of 3-hinges. Cortical parcellations at the resolution of 500 patches on each hemisphere were used to construct the structural connective networks. **b** Mean nodal graphic metrics comparison among convolution patterns within species. The metrics were normalized via *z*-score transformation and the mean values were averaged over subjects. * indicates *p* value of *t*-test is equal or smaller than 0.05. ** indicates *p* value of *t*-test is equal or smaller than 0.01. *** indicates *p* value of *t*-test is equal or smaller than 0.001. Abbreviations: M: macaque, C: chimpanzee

In Fig. 4a, we present the spatial distribution of the nodal metrics of the structural network by mapping the metrics to white matter surfaces. Local maximums of these metrics are frequently found in the locations where 3-hinges reside. These observations were further confirmed by statistical analyses shown in Fig. 4b, where the discrepancies between *h3* and *h2* are significant (low *p*-values of the two-sample *t*-tests) within the two species. There are two exceptions: betweenness on macaque brains and clustering coefficient on chimpanzee brains. Similar to experiments on human brains, random permutation tests were also conducted. All *p*-values were below 0.05, confirming that the difference between 3-hinges and 2-hinges on chimpanzee and macaque brains was not produced by chance. The permutation tests were not performed on the two exceptions mentioned above. It is noted that the *h3*-*h2* contrast in efficiency and clustering coefficient between these two species does not reproduce those on human. On these two species, 3-hinges exhibit higher values in these two metrics, suggesting that their 3-hinges have more direct connections to densely self-connected modules.

We conducted a comparative study among the three species to investigate that if the contrast between 3-hinges and 2-hinges is a simple replication from macaque brains to human brains or is different across species. It is noted that the cross-subject *z*-score transformation was performed for the metrics within each species. The mean values (± standard deviations) for 3-hinges and 2-hinges averaged over subjects within each species are reported in the 1st section of Table 4. We conducted *t*-tests (two-tail) between species pairs for each of the convolution patterns to see if the metrics were significantly different across species. The *p*-values are reported in the 2nd section of Table 4. The metric values decrease from human brains to macaque brains for both 3-hinges and 2-hinges in degree, strength, betweenness and participation, while increasing in efficiency and clustering coefficient. The significance of the cross-species difference was confirmed by low *p*-values for most *t*-tests. Insignificant difference was mostly found between chimpanzee and macaque (bold type).

**Table 4:** Section 1: The mean graphic metrics (± standard deviations) of 3-hinges and 2-hinges; Section 2: *P*-values of two sample *t*-tests for the graphic metric differences between species on each cortical folding pattern

		DEG	STR	BET	EFF	CLU	PAR
Section 1							
h3	H	0.55 ± 1.20	0.59 ± 1.28	0.37 ± 1.44	−0.06 ± 0.83	−0.05 ± 0.77	0.18 ± 0.97
	C	0.46 ± 1.26	0.53 ± 1.43	0.34 ± 1.35	0.19 ± 0.98	0.04 ± 0.89	0.10 ± 0.96
	M	0.14 ± 0.98	0.18 ± 1.14	0.07 ± 0.91	0.21 ± 1.10	0.15 ± 1.04	0.05 ± 0.99
h2	H	0.14 ± 0.93	0.10 ± 0.92	0.06 ± 0.92	−0.03 ± 0.92	−0.01 ± 0.89	0.12 ± 0.98
	C	0.07 ± 1.02	0.07 ± 1.02	0.06 ± 1.02	0.06 ± 1.03	0.03 ± 1.01	0.02 ± 0.98
	M	0.08 ± 1.04	0.09 ± 1.12	0.05 ± 1.04	0.09 ± 1.05	0.06 ± 1.03	−0.02 ± 1.00
Section 2							
h3	H vs. C	4.20 × 10^−3^	0.036	**0.45**	7.70 × 10^−31^	9.83 × 10^−6^	3.77 × 10^−6^
	H vs. M	1.54 × 10^−23^	3.11 × 10^−20^	8.28 × 10^−10^	5.71 × 10^−20^	1.84 × 10^−13^	2.78 × 10^−12^
	C vs. M	1.99 × 10^−11^	2.40 × 10^−10^	3.14 × 10^−8^	**0.86**	5.38 × 10^−3^	3.39 × 10^−2^
h2	H vs. C	4.82 × 10^−6^	0.05	0.89	1.90 × 10^−11^	1.51 × 10^−3^	3.40 × 10^−8^
	H vs. M	1.70 × 10^−3^	**0.83**	0.80	2.69 × 10^−11^	6.42 × 10^−5^	3.65 × 10^−12^
	C vs. M	**0.80**	**0.34**	**0.84**	**0.29**	**0.26**	4.92 × 10^−2^

The metrics were z-score transformed across subjects within each species.

*Abbreviations*: *h3* 3-hinge, *h2* 2-hinge, *H* human, *C* chimpanzee, *M* macaque, *DEG* degree, *STR* strength, *BET* betweenness, *EFF* efficiency, *CLU* clustering coefficient, *PAR* participation

**Fig. 5:**
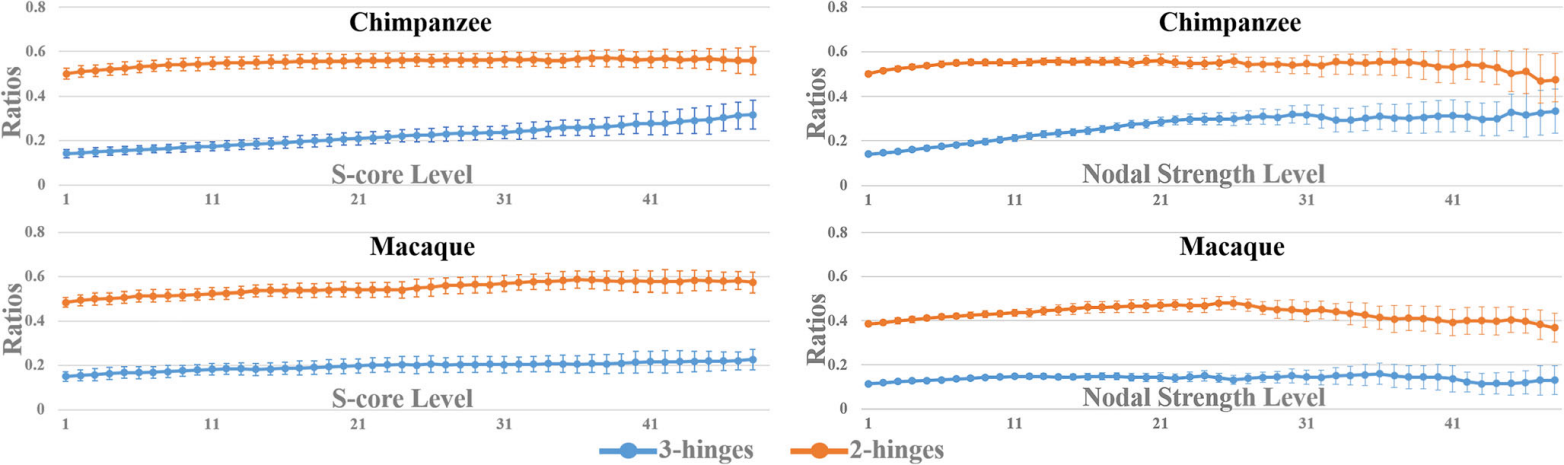
Network core studies on chimpanzee and macaque brains. Left: Ratios of numbers of preserved 3-hinges and 2-hinges at an *s*-core level, at which edges with strength less than *s* were deleted. Right: Ratios of numbers of preserved 3-hinges and 2-hinges at a nodal strength level, at which nodes with strength less than *s* were deleted. The values were averaged over subjects and the bars indicate the standard deviations

We also performed analyses on network cores for the two species. In general, it is found in Fig. 5 that the network core recruits increasingly more 3-hinges at higher core levels on the two species. However, unlike the result on human brains that 3-hinge number surpasses that of 2-hinge at higher core levels (black arrow heads in Fig. 3b and c), the ratio curves don’t cross each other on chimpanzee brains and macaque brains. On chimpanzee brains, the two curves keep getting closer and the 3-hinge ratio approaches 0.3 at the curve tails for both decomposition methods (which is around 0.6 on human). These two ratio curves, however, appear to be parallel lines on macaque brains, and the 3-hinge ratio slowly approaches 0.2. The different trends of the ratio curves on the three species suggest that more 3-hinges are involved in the high-level core of structural cortico-cortical connective networks on higher species.

## Discussion and conclusion

In this work, we subdivide cortical gyrus to 3-hinges and ordinary gyri (2-hinges) according to their folding morphology. The subdivision could possibly reflect and quantify the intrinsic difference between these cortical folding patterns. The roles different folding patterns play in the brain networks are the major focus of this work.

In general, we found that 3-hinges possess higher degrees, strengths and betweennesses than 2-hinges on human brains. It has been suggested in previous studies that gyri could serve as cortical hubs in contrast to sulci (Deng et al. 2014; Jiang et al. 2015). The difference between 3-hinges and 2-hinges in this work suggests that gyri could be sub-divided and there could be a hierarchical organization within the gyral system. The higher participation coefficients of 3-hinges, associated with their higher degrees, strengths and betweennesses, suggest that 3-hinges could be more like connector hubs between cortical communities rather than provincial hubs with communities. Lower efficiency values and clustering coefficients of 3-hinges support this hypothesis by showing that the neighboring nodes of 3-hinges are more sparsely connected. At a more global scale, the core of the structural network could include more 3-hinges than 2-hinges, and such a discrepancy was found to grow with the increasing core-level. These structure-based results were well supported by our task functional MRI data analysis, in which we found that 3-hinges were simultaneously involved in more global functional networks than 2-hinges.

These abovementioned local-scale differences between 3-hinges and 2-hinges are reproduced on both chimpanzee brains and macaque brains. However, 3-hinges on these two species do not behave like connector hubs as pronounced as those on human brains, due to their larger efficiency, clustering coefficient and lower proportions in higher-level cores. The cross-species consistency and variability are discussed later.

### Relation between cortical folding, structure and function

The relation between cortical convolution, anatomical connections and brain function have been investigated for decades (Connolly 1950.; Richman et al. 1975; Rakic 1984; Van Essen 1997; Hilgetag and Barbas 2005). The roles of different cortical folding patterns in cortico-cortical networks have to be associated with their architectonics. In previous studies that focused on gyrus-sulcus pairs, it has been suggested that gyri differ from sulci in neuron number and dendrite morphology (Hilgetag and Barbas 2005). These micro-scale differences could provide an intrinsic interpretation for the macro-scale difference between gyri and sulci. For example, thicker cortices of gyri may be related to more neurons in them (Hilgetag and Barbas 2005). Along this line, we might posit that 3-hinges have more neurons and that the distribution and morphology of dendrites and axons could also be different between 3-hinges and ordinary gyri.

**Fig. 6:**
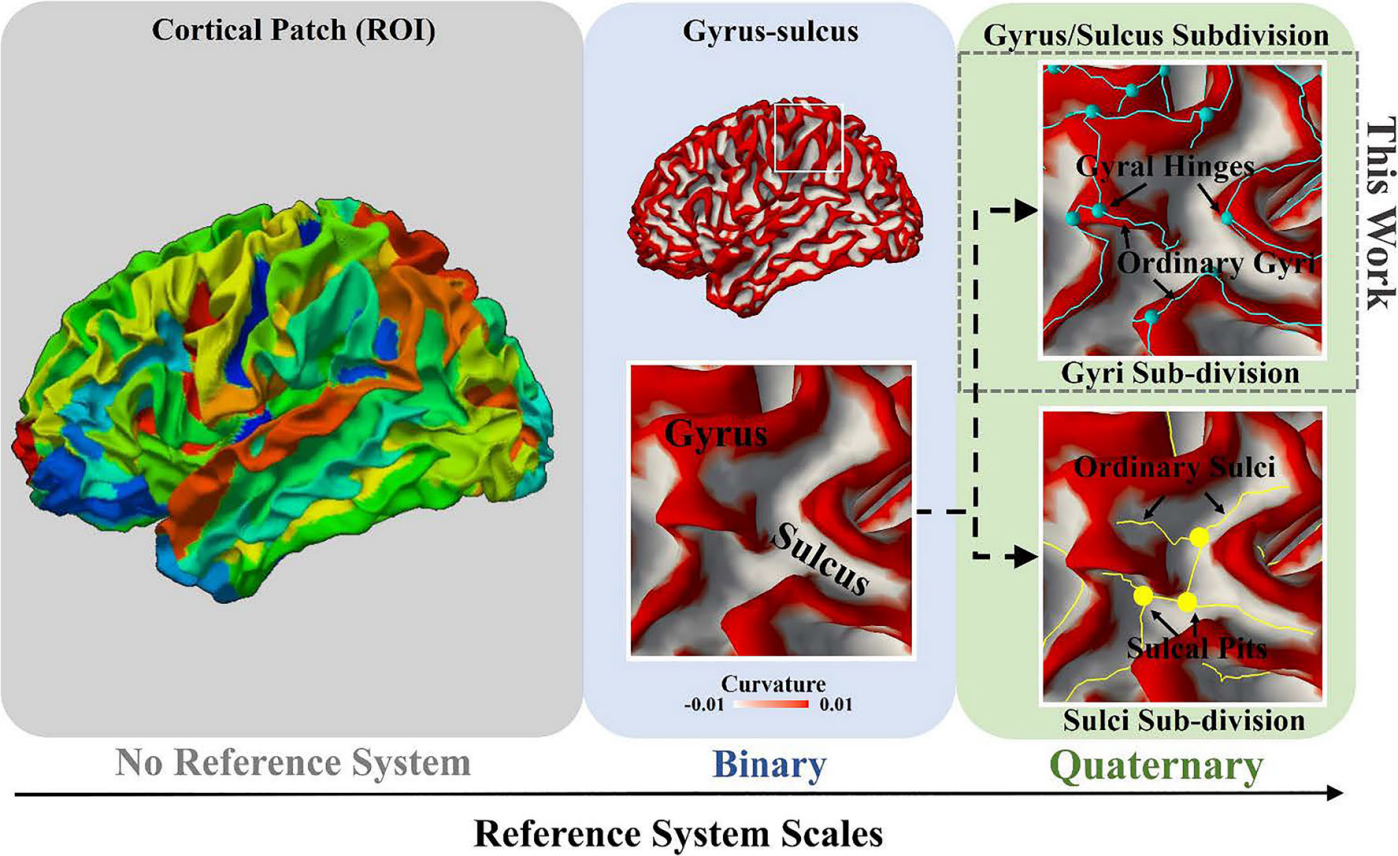
The approaches of cortical folding analyses at different scales. The dashed frame highlights where this work belongs. Interpretations are found in the texts

At a macro-scale, cortical convolution patterns were demonstrated to be predictive of cortical regions of different cyto-architectures (Fischl et al. 2007) in primary cortex. The concept of structural ‘connectional fingerprint’ was proposed and has been demonstrated to be unique for each cortical area and underlies the associated ‘functional fingerprint’ (Passingham et al. 2002). Based on these previous conclusions and our findings, we could intuitively posit that the cortical folding patterns are correlated to the brain functions. However, at the resolution of gross brain areas (the leftmost panel in Fig. 6), such as BAs, folding patterns seem to be too variable to be competent for a function predictor (Fischl et al. 2007). This was demonstrated by relatively poor performance by using stereotaxic coordinate system (Talairach et al. 1967; Talairach and Tournoux 1988) and cortical morphological descriptors, such as the gyrification index (Zilles et al. 1997; Schaer et al. 2008), as predictors of cortical areas of higher brain functions. While the reliability of cross-subject analyses is subject to the precision of brain alignment algorithms, a lack of a cortical folding reference system could be another critical reason for the poor performance. Without it, these cortical morphological descriptors could only provide a gross measurement for a large cortical patch.

In contrast to cortical-area-based analyses, the cortex was usually divided to gyri and sulci. This division provides a binary cortical folding reference system, based on which a cortical area can be decomposed to two basic units (the middle panel of Fig. 6). The effectiveness of this gyrus-sulcus reference system was demonstrated by the different cyto- and myelo- architectures between the two basic units (Connolly 1950; Richman et al. 1975; Rakic 1984; Van Essen 1997; Hilgetag and Barbas 2005). Also, there is a consensus that different functional roles could be played by gyri and sulci, though it is still controversial that how the patterns of architectonics are different between gyri and sulci (Van Essen 1997; Xu et al. 2010; Nie et al. 2012; Chen et al. 2013; Budde and Annese 2013; Zhang et al. 2014) and how to infer neuronal and cortical functions from the different patterns (Van Essen 1997; Deng et al. 2014; Jiang et al. 2015). Therefore, a better understanding of how a cortical area is organized within this gyrus-sulcus reference system might help accurately define a cortical area on different individuals and to make a comparison among them. This individualized analysis might not be subject to brain alignment accuracy, and thus might facilitate the cross-subject comparison, and further enhance the reliability of the structure-function relation inferred from them. However, the principle that how a cortical area is organized by gyri and sulci is not clear by far. In our previous studies (Deng et al. 2014; Jiang et al. 2015), gyri were suggested to serve more like hubs than sulci in cortico-cortical networks. This conclusion was drawn based on a whole-brain-scale gyrus-sulcus contrast. But when the cortex was parcellated, this gyrus-sulcus contrast was not always as pronounced on one cortical region as the one on another (Jiang et al. 2015). This observation suggests that one gyrus-sulcus organization principle on a cortical region is not guaranteed to apply to another. Another possible reason is that the gyrus-sulcus reference system might not be accurate and basic enough. They can be further decomposed to more basic folding units (The rightmost panel in Fig. 6) with a finer granularity. In many previous studies, sulcal pits, the locally deepest regions on sulcal regions, were demonstrated to be more consistent across subjects than other sulcal regions and to provide an organizing framework for cortical folding (Lohmann et al. 2007; Auzias et al. 2015; Im and Grant 2019). Similar observations were found in our gyrus-centered studies including this work. The way that 3-hinges are different from other gyri seems similar to that sulcal pits are from other sulci. In fact, we have manually identified a group of 3-hinges that possess cross-subject correspondences (Li et al. 2017). These correspondences can even be established across species (Li et al. 2017). Therefore, it is reasonable to expect that 3-hinges provide an organizing framework for the gyrus system (Chen et al. 2017), as what was suggested for sulcal pits in Lohmann et al. 2007. In this respect, we could posit that the gyrus-sulcus reference system is sub-dividable to a quaternary system as illustrated in Fig. 6 (the rightmost panel). A hierarchy could thus be hypothesized for cortical folding patterns, where sulcal pits and 3-hinges demonstrate higher cross-subject consistency than the other two units: ordinary sulci and ordinary gyri. In this hierarchy, 3-hinges have also been demonstrated in this work to serve more like connector hubs while the ordinary gyri serve more like provincial hubs, and it is expected that sulcal pits and ordinary sulci find their different positions in the network hierarchy as well. It is hoped that this quaternary system provides a much finer and more accurate reference, such that more profound clues can be found for the principle of cortical organizations and the structure-function relations. With no doubt, a further sub-division on this quaternary system can be performed and the resolution can definitely be pushed to even finer levels.

### Parsimonious principle of wiring cost in brain development

Cortical folding patterns are the mixed results of multiple developmental processes, such as neuron migration, neuron proliferation, axonal projection and pruning. Therefore, gyro-sulcal patterns’ anatomical and functional roles were usually associated with gyro-sulcal patterns’ developmental mechanisms. For example, in Van Essen 1997, by assuming axons are under tension, they asserted that axons pull the cortical regions with denser connections closer to each other, resulting in convex and concave folding patterns. More importantly, by pulling the cortical regions closer to each other, these shortened axons could increase the information transit efficacy. This hypothesis suggested an anatomical wiring diagram that was controlled by parsimonious principle of wiring cost (Ramón y Cajal 1995; Kaiser and Hilgetag 2004; Kaiser and Hilgetag 2006; Garcia-Lopez et al. 2010; Rubinov et al. 2015). Another group of studies found that axons are radially distributed in the convex folds (gyri) while circumferentially course along the deep boundaries of the concave folds (sulci) (Xu et al. 2010; Nie et al. 2012; Budde and Annese 2013; Chen et al. 2013; Zhang et al. 2014). This organization is seemingly irreconcilable with the parsimonious principle of wiring cost, as the densely connected regions (gyri) are far from each other. But the cortical areas are not connected with equal chance. For example, small-world and rich-club features have been discovered on the brain wiring diagram (a full cover of review is found in Bullmore and Sporns 2012) to emphasize the extra importance of some particular regions, which are usually defined as hubs with higher nodal degrees and comprise a densely self-connected core in the network. In this work, 3-hinges only take up a small portion of the gyral system, but possess an unmatched large portion of the total structural connective strengths (Figs. 3b and c, 5 and Fig.S5). This discrepancy is in line with the organization principle in small-world and rich-club networks that large network resources concentrate on only a small portion of nodes (Harriger et al, 2012). Therefore, in this group of theories, it is inferred that the information transit efficacy could be maximized by optimizing the cortical network organization in a hierarchical manner at a more global scale rather than by locally shrinking the axon length between only two areas. Finally, with regard to our hypothesized quaternary reference system (the rightmost panel in Fig. 6), the spatially consistent distributions of sulcal pits across individuals have already been reported to be present even at birth and remain stable during the early development phase (Meng et al. 2014; Im and Grant 2019). 3-hinges, as folding patterns coupled with sulcal pits, are expected to appear early and remain stable as well. The brain seems to give priority to develop a minority of functionally more important cortical regions.

### Cross-species comparison and abnormality

In general, the cross-species difference is more significant for 3-hinges (Table 4) and is less significant for 2-hinges especially between chimpanzee and macaque. The higher significance in 3-hinge might suggest that the differentiation on the higher-order hubs (3-hinges, more like connector hubs) is becoming pronounced across species, while the function of the secondary hubs (2-hinges, more like provincial hubs) could be similar. The growth in absolute numbers of 3-hinges from macaque to human implies the need for more information gathering and distributing centers to deal with more advanced tasks. The growth in 3-hinges’ graphic metrics from macaque to human (Table 4, except efficiency and clustering coefficient) implies the upgrading competence of 3-hinges which could handle interactions of more sub-networks on higher order species. For example, by comparing the regions with peak graphic metric values across species in Figs. 3a and 4a (note that the maps are not comparable across species because of different value scales, but the spatial distribution of peak-value regions are comparable), we can observe a progressive extension of peak values to temporal, and parietal association cortical areas, which are the phylogenetically late-developing areas and correspond to the human-specific elaboration of cognitive functions (Goldman-Rakic 1988; Kaas 2006; Rakic 2009; Smaers et al. 2011). The participation coefficients of 3-hinges are higher than 2-hinges on all species, suggesting that the roles of 3-hinges as connector hubs could be preserved across species. However, the inverse h3-h2 contrast in efficiency and clustering coefficient was found between macaque/chimpanzee and human. A possible interpretation is that connector hubs on human brains could be organized in a hierarchical manner. Some connector hubs could be directly linked to modules such that they have higher efficiency values and clustering coefficients. These connector hubs could be the primary ones. For those having low efficiency and clustering coefficient (that is, their direct neighbors are sparsely connected), they could be secondary connector hubs that directly link the primary connectors (primary connector hubs could be sparsely connected within themselves).

Since brains within the same mammalian order (such as primates) become more convoluted as a function of mass, it is not surprising that 3-hinges will be more common in humans than in chimpanzees, and more common in chimpanzees than in macaques. However, the different cortical areas do not uniformly expand as a function of their size. Some regions, such as the superior temporal gyrus, ventrolateral prefrontal cortex and anterior cingulate cortex, expand far more steeply than, for example, the inferior temporal and parahippocampal regions (e.g. Chaplin et al. 2013). If the 3-hinges were connectivity hubs that correlate with the degree of behavioral complexity, one would expect that 3-hinges would be more common in such regions which are particularly distinctive in human brains. In fact, 3-hinges are not uniformly distributed across the brains (Zhang et al. 2018). For example, the frontal lobe of human has 37.78 ± 0.51% of cortical areas but 38.32 ± 2.98% of 3-hinges (Fig. S5). In contrast, the frontal lobe of macaque has 29.07 ± 1.11% of cortical areas but 28.92 ± 0.92% of 3-hinges. Such a discrepancy can be found on all the other lobes and becomes larger from human to macaque.

Finally, associating the anatomical and functional networks with cortical folding patterns could also be helpful to study brain abnormalities and lesions. Many brain diseases are accompanied by abnormal cortical folding patterns (Thompson et al. 2004; Nordahl et al. 2007). If different folding patterns play different roles in brain networks, the anatomic locations of abnormalities and lesions will induce different levels of brain disorders. For example, abnormalities or lesions on 3-hinges could have a higher change to injure hubs, which could induce malfunction of distributed areas rather than an isolated one (Liu et al. 2008; Lynall et al. 2010; Fornito et al. 2015).

### Limitations

The observation and implication of this work are largely based on dMRI datasets. The accuracy of fiber orientation estimation from dMRI at the interface of white matter and gray matter could be biased by the curvedness of cortical surface, known as ‘gyral bias’, making it difficult to track streamlines penetrating the boundaries between gray matters and white matters at the sulcal regions. Therefore, we limit our interest in the folding patterns within the gyral regions to circumvent the ‘unfair’ and ‘biased’ comparative results. Even so, the results could be biased due to other known limitations of dMRI. For example, the low spatial resolution results in uncertainty in voxels where fiber fanning, crossing and kissing are pronounced. Spurious pathways could be estimated, because orientation models and tractography algorithms are sensitive to parameters and noises. Also, it is incapable of identifying the short-range axonal pathways such as those within the cortex and those in superficial regions of white matters (Reveley et al. 2015). Therefore, the results and hypotheses in this work need evaluations by means of more ‘direct’ but invasive techniques, such as histology and tract-tracing in animal models. For instance, since the 3- and 2- hinges exist in macaque brains, we could examine if different numbers of labelled neurons after retrograde injections are observed in these macroscopic structures. These nontrivial future endeavors will shed more light to tackle the aforementioned imaging issues. Finally, to estimate functional networks, we adopted our earlier home-made method (Jiang et al. 2015) which is based on dictionary learning and sparse representation. This method is easy to be applied on other datasets. The inferred functional networks are easy to interpret. However, the model is shallow, making the inferred networks may not be as faithful as those inferred from deep models. We believe that many promising deep-learning-based methods (Cui et al. 2018; Zhao et al. 2018; Dong et al. 2019) deserve more efforts.

## Electronic supplementary material

ESM 1 (DOCX 1122 kb)

## Footnotes

1 https://www.humanconnectome.org/software/connectome-workbench
