# Supplementary material for: Cortical 3-hinges could serve as hubs in cortico-cortical connective network
Source: Brain Imaging Behav. 2020 Jan 16;14(6):2512–29. doi: 10.1007/s11682-019-00204-6 (PMC7647986; doi:10.1007/s11682-019-00204-6)
Supplement: Supplementary file 1 — (DOCX 1122 kb) [file 11682_2019_204_MOESM1_ESM.docx]

**Cortical 3-hinges Could Serve as Hubs in Cortico-cortical Connective Network**

**Brain Imaging and Behavior**

Tuo Zhang^1*^, Xiao Li^1*^, Xi Jiang^2^, Fangfei Ge^3^, Shu Zhang^3^, Lin Zhao^1^, Huan Liu^1^, Ying Huang^1^, Xianqiao Wang^4^, Jian Yang^5, 6^, Lei Guo^1^, Xiaoping Hu^7^, Tianming Liu^3^

^1^School of Automation, Northwestern Polytechnical University, Xi’an, China; ^2^The Clinical Hospital of Chengdu Brain Science Institute, MOE Key Lab for Neuroinformation, School of Life Science and Technology, University of Electronic Science and Technology of China, Chengdu, China; ^3^Cortical Architecture Imaging and Discovery Lab, Department of Computer Science and Bioimaging Research Center, The University of Georgia, Athens, GA, USA; ^4^College of Engineering, The University of Georgia, Athens, GA, USA; ^5^Radiology Department of the First Affiliated Hospital, Xi'an Jiaotong University, Xi'an, China; ^6^The Key Laboratory of Biomedical Information Engineering, Ministry of Education, Department of Biomedical Engineering, School of Life Science and Technology, Xi’an Jiaotong University, Xi’an China; ^7^Department of Bioengineering, University of California Riverside, Riverside, CA, USA.*Joint first authors. #Corresponding author, address: #127, West Youyi Road, Xi’an, Shaanxi, China, 710072; Telephone: (+86)17795741808; Email: tuozhang@nwpu.edu.cn


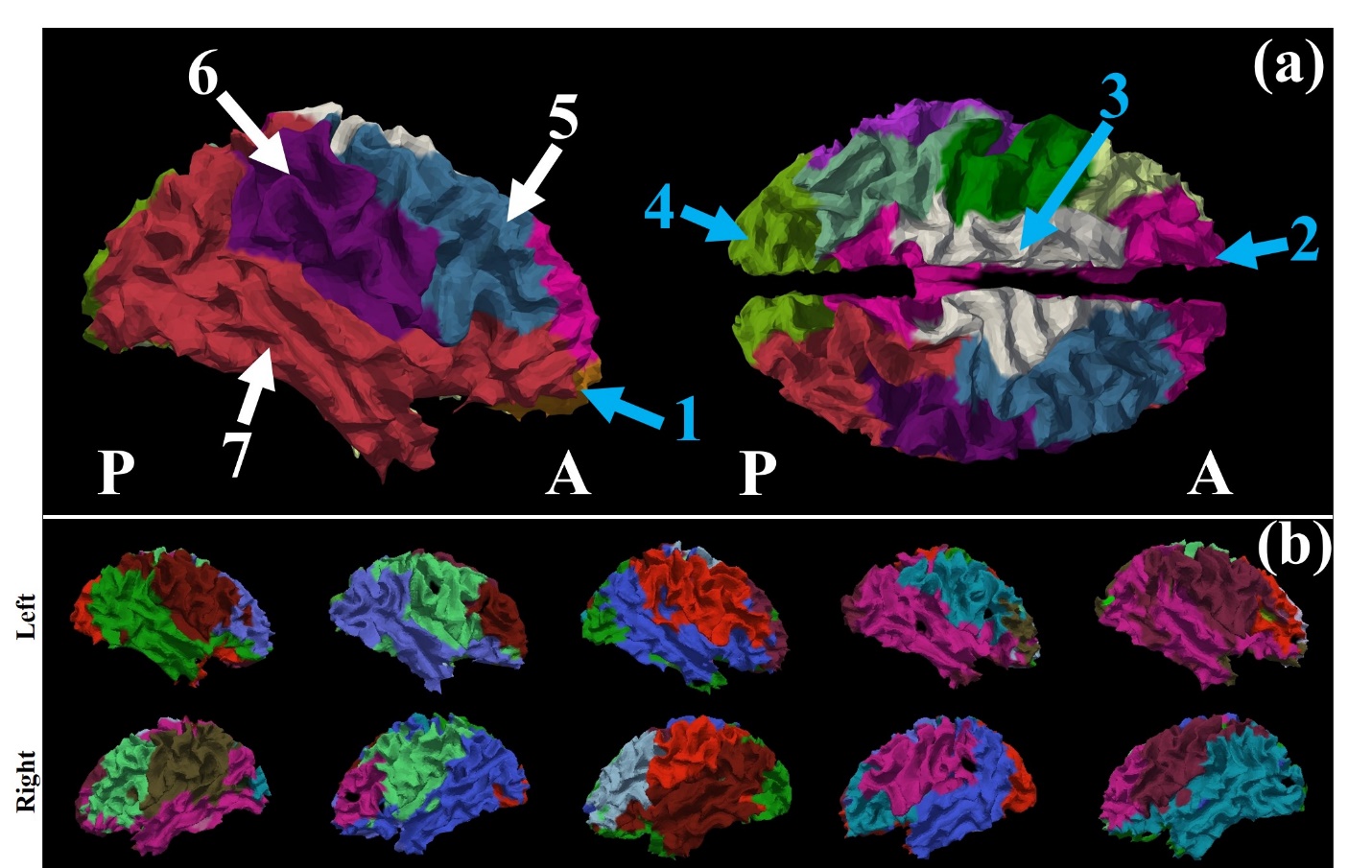


Fig. S1 (a) Modules of a structural connective network of an example subject via the data-driven method in Brain Connectome Toolkit (https://sites.google.com/site/bctnet/). 1: Orbital-frontal cortex. 2: Cingulate & frontal lobe. 3: Dorsal somatomotor network. 4: Visual cortex. 5: Frontal cortex & motor cortex. 6: Ventral somatomotor network. 7: Visual association cortex, auditory network and frontal-parietal cognitive control network. Modules in 5-7 find their counterparts on the other hemispheres. (b) The modules on five randomly selected subjects. It is noted that the module partition is separately performed on each subject. Therefore, no cross-subject correspondence of these modules are strictly identified (different color scales on subjects) and the module numbers are slightly different, but cross-subject consistency on major modules can be observed


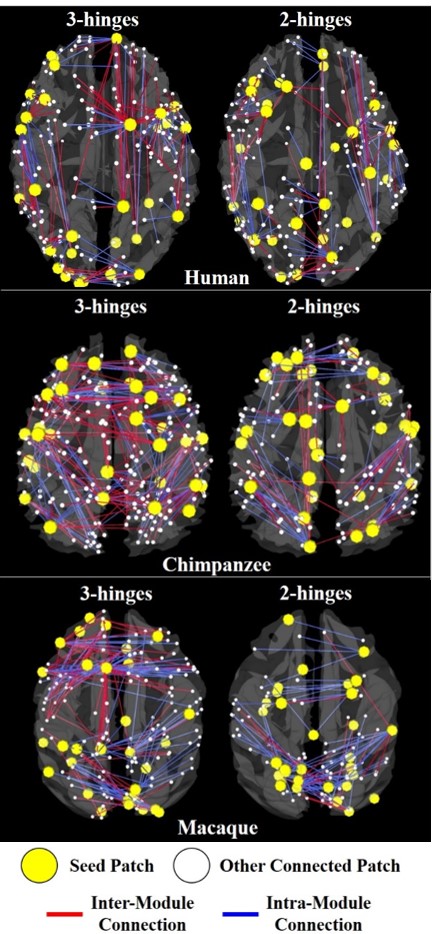


Fig. S2 The roles 3-hinges and 2-hinges play among cortical modules. 30 3-hinge patches (yellow dots left in each sub-figure) and 30 2-hinge patches (yellow dots right in each sub-figure) were randomly selected as seeds for the ease of visualization. Lines represent the connections (edges in the connective matrix) derived from these seeds and the other connected seeds are represented by white dots. Red lines are inter-module connections and blue lines are intra-module ones. It is observed that the 3-hinge derived connections have more inter-module connections than intra-module ones (more red lines in the left columns of each sub-figure), while the opposite observation is found for the 2-hinges (more blue lines in the right columns)


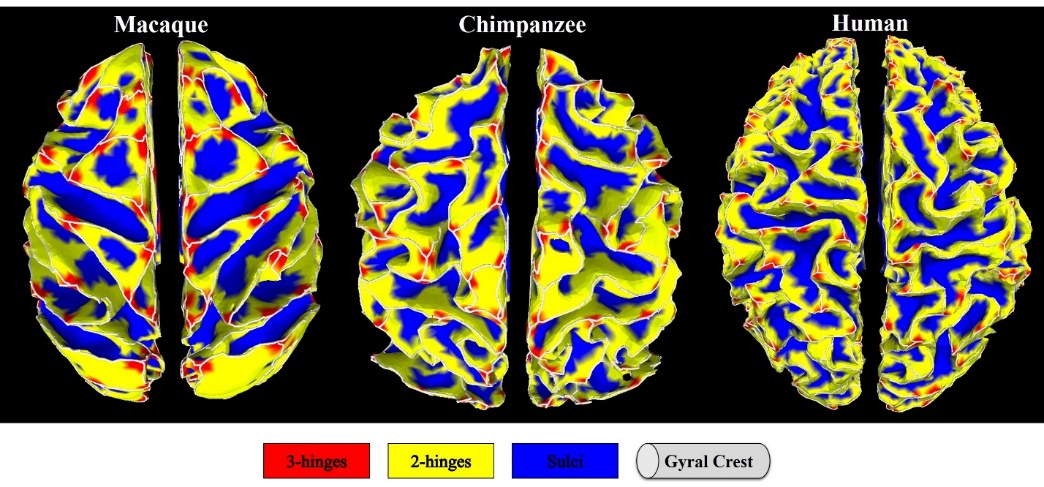


Fig. S3 3-hinge, 2-hinge and sulcal cortices on example subjects of three species. White curves are the gyral crest lines. Locations of 3-hinges are highlighted by red color


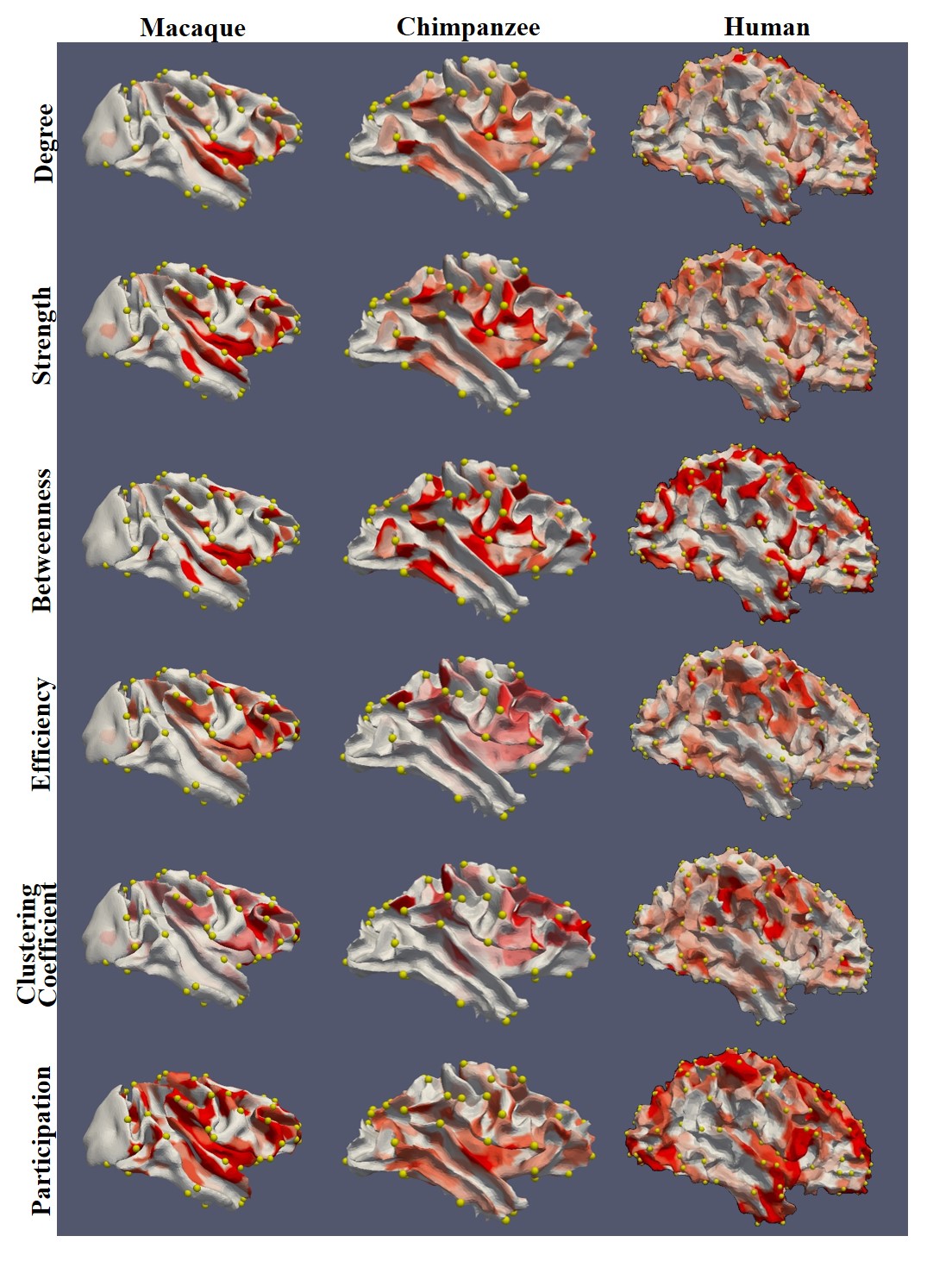


Fig. S4 Graphic metrics of structural connective networks mapped to white matter cortical surfaces of the right hemispheres of the three species. For each metric, red color indicates high value and white indicates low value. Color bar for each map was individually tuned for better result visualization. Thus, the color maps in this figure are not comparable across species (The value scales are referred to Figs. 3&4 in the main texts). For each species, one subject is randomly selected as the illustrative example. Yellow bubbles highlight the locations of 3-hinges


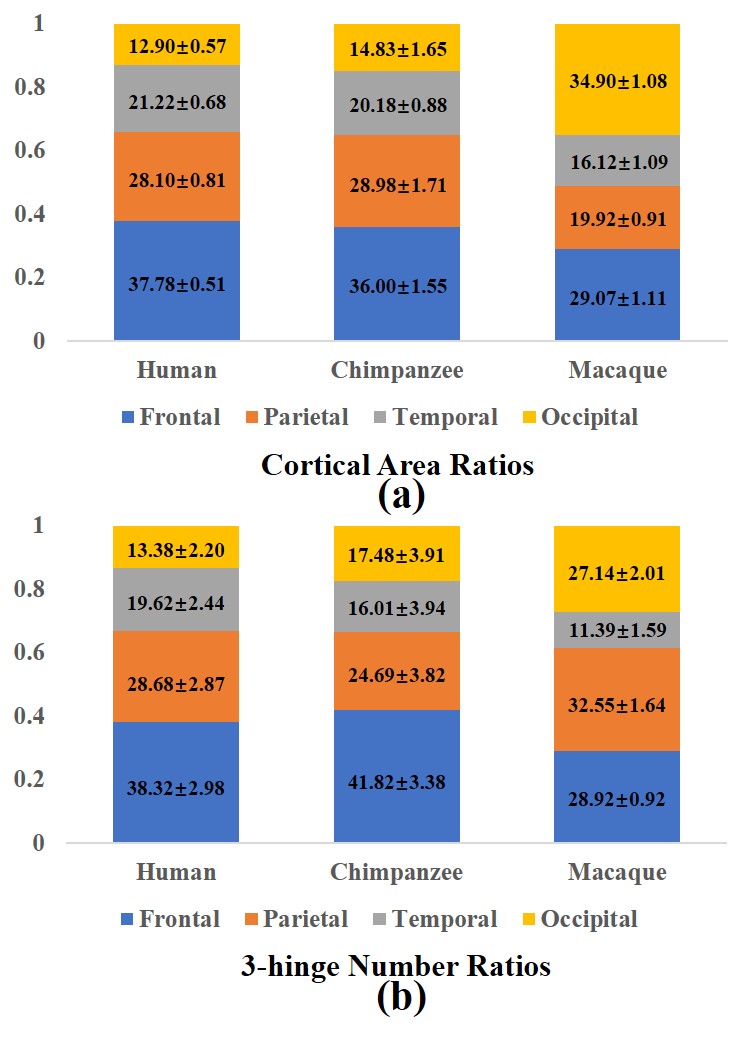


Fig. S5 Cross-species comparison. (a) Cortical area ratios of lobes; (b) 3-hinge number ratios of lobes. Only frontal lobe, parietal lobe, temporal lobe and occipital lobe are considered
